# Assessing the feasibility and sustainability of a surfactin production process: a techno-economic and environmental analysis

**DOI:** 10.1007/s11356-024-32217-0

**Published:** 2024-04-09

**Authors:** Johnny Alejandro Poveda-Giraldo, Juan Camilo Solarte-Toro, Chantal Treinen, Philipp Noll, Marius Henkel, Rudolf Hausmann, Carlos Ariel Cardona Alzate

**Affiliations:** 1 Departamento de Ingeniería Química, Universidad Nacional de Colombia Sede Manizales, Instituto de Biotecnología y Agroindustria, Km 07 Vía Al Magdalena, Manizales, Colombia; 2 Cellular Agriculture, TUM School of Life Sciences, Technical University of Munich, Gregor-Mendel-Str. 4, 85354 Freising, Germany; 3 Institute of Food Science and Biotechnology, Department of Bioprocess Engineering (150k), University of Hohenheim, Fruwirthstr. 12, 70599 Stuttgart, Germany

**Keywords:** Process simulation, Surfactin, Bioprocess engineering, Life cycle assessment, Winter wheat, Carbon footprint

## Abstract

Biosurfactants have been profiled as a sustainable replacement for chemical-based surfactants since these bio-based molecules have higher biodegradability. Few research papers have focused on assessing biosurfactant production to elucidate potential bottlenecks. This research aims to assess the techno-economic and environmental performance of surfactin production in a potential scale of 65m^3^, considering different product yields and involving the European energy crisis of 2021–2022. The conceptual design, simulation, techno-economic, and environmental assessments were done by applying process engineering concepts and software tools such as Aspen Plus v.9.0 and SimaPro v.8.3.3. The results demonstrated the high economic potential of surfactin production since the higher values in the market offset the low fermentation yields, low recovery efficiency, and high capital investment. The sensitivity analysis of the economic assessment elucidated a minimum surfactin selling price between 29 and 31 USD/kg of surfactin, while a minimum processing scale for economic feasibility between 4 and 5 kg/h is needed to reach an equilibrium point. The environmental performance must be improved since the carbon footprint was 43 kg CO_2_eq/kg of surfactin. The downstream processing and energy demand are the main bottlenecks since these aspects contribute to 63 and 25% of the total emissions. The fermentation process and downstream process are key factors for future optimization and research.

**Supplementary Information:**

The online version contains supplementary material available at 10.1007/s11356-024-32217-0.

## Introduction

Biosurfactants are key molecules used in cosmetic, pharmaceutical, petroleum, agricultural, food, and chemical industries since these components can reduce surface and interfacial tension (Aslam et al. 2021; Banat et al. 2021). These molecules can be classified as specialty chemical because biosurfactants are produced at lower scales than bulk chemicals (Fabbri et al. 2018; Sarubbo et al. 2022). Biosurfactants have an amphiphilic character (i.e., molecules with hydrophilic and hydrophobic parts), making these molecules suitable components in end-products (Sarubbo et al. 2022). For instance, biosurfactants are used in the agricultural industry as an addition to herbicide formulations and control phytopathogens (Ramesh and Abinaya 2022). Moreover, these molecules have also been applied to make soil bioremediation by recovering oil from contaminated soils (Parus et al. 2023). Biosurfactants have been characterized as environmentally friendly molecule due to low toxicity, high compatibility, and fast biodegradability (Markande et al. 2021). Biosurfactants have been used as ingredients in medicines because of their anti-microbial, antitumor, antiviral, and immune-modulating characteristics (Ramalingam et al. 2019; Ambaye et al. 2021). Further promising biosurfactant applications are related to producing household detergents and personal care products since these compounds are the active ingredients (Lee et al. 2018). Indeed, the biosurfactants market size is expected to grow from 4.18 to 6.04 billion USD in 2022–2029 (Insights Business 2022). Thus, biosurfactants will exhibit a compound annual growth rate (CAGR) of 5.4% during the forecast period (Insights Business 2022). The most important actors and potential customers of this relevant value chain are Dow Inc, Allied Carbon Solutions CO Ltd, Saraya Co Ltd, Evonik Industries AG, BASF SE, Sabo SPA, Holiferm Ltd., Stepan Co., Deguan Biosurfactant Supplier, Jeneil Bioproducts GmbH, Biofurture, Ecochem, Synthesize LLC, AGAE Technologies, TeeGene Biotech, Ecover, and Kaneka Co. (Fabbri et al. 2018; Insights Business 2022).

Biosurfactant production faces important challenges before replacing chemical surfactants (Taghavi et al. 2022). The most important challenges are related to techno-economic issues. Technical problems are related to low production yields, low product concentration, high mass intensity, and high specific energy demand (Kanawal et al. 2021; Gaur et al. 2022). Economic issues are related to biosurfactant separation and purification since technologies such as foam fractionation, membranes, gravity separation (i.e., acid precipitation, crystallization), and ultrafiltration have high capital and operating costs (Aslam et al. 2021). These problems have limited the substitution of chemical surfactants worldwide. Biosurfactants can be classified as glycolipids, phospholipids, polymeric biosurfactants, and lipopeptides (Czinkóczky and Németh 2020). Surfactin, a lipopeptide, can be produced using different carbon sources (i.e., glucose, xylose, and sucrose). *Bacillus subtilis* has been studied as a potential microorganism to produce surfactin. Nevertheless, advances such as high-density fermentation need to be scaled up to encourage commercial applications (Klausmann et al. 2021). Few studies have analyzed techno-economic aspects related to the surfactin production process. Czinkóczky and Németh (2020) analyzed different plant configurations to produce surfactin and lichenysin, assuming 0.16 and 0.024 g/g of glucose as yields. These authors conclude that the simultaneous production of these molecules can be feasible at scales higher than 18.2 tonnes per year. Nevertheless, these authors left aside the environmental impact of the process.

Potential process improvements based on metabolic engineering, bioprocess engineering, chemical engineering, and process engineering have been proposed. Indeed, genetically modified microorganisms (GMOs) have been designed to increase product-to-substrate yields (Markande et al. 2021). Hybrid production schemes, which integrate product formation and separation, have been studied to reduce processing steps and production costs. Nevertheless, these options have been developed at the research level (Technological Readiness Level—TRL 1 to 3) (Dolman et al. 2019). In addition, few techno-economic and environmental studies based on the conceptual design of the biosurfactant production processes have been reported to elucidate potential technologies for scale-up. However, biosurfactant development should be beyond the academic sector. The industrial development of biosurfactants, especially surfactin, is linked with establishing a circular bioeconomy because this high-value product can be synthesized using renewable sources (Koul et al. 2022). The development of biosurfactants is increasing based on current government laws/policies that regulate the use of environmentally harmful chemicals. These laws/policies have been announced and implemented worldwide, further fostering the transition towards the use of eco-friendly and sustainble products on the part of the manufacturers (Nagtode et al. 2023). For instance, BASF SE has signed partnership agreements with ACS (Allied Carbon Solutions Co., Ltd) and Holiferm Ltd to focus on advancements in sustainable biosurfactant production for the personal and home care sector. The actions carried out by BASF can serve as an example to encourage the production of bio-based products in agreement with the policies, laws, and financial incentives (Wesche and Hellmann 2021). The research gap addressed in this paper is related to the technical, economic, and environmental information about biosurfactant production. This statement is raised based on academic and industrial trends presented above. This research study seeks to contribute to the literature related to biosurfactant production, aiming to elucidate more options, strategies, and ideas for increasing bio-based product generation.

The statement of the novelty of this research study is related to the integral assessment of the surfactin production process using glucose as substrate produced from a renewable raw material as feedstock (i.e., winter wheat) since most research papers use raw glucose as feedstock leaving aside the pretreatment stage of biomass sources (one of the most energy-demanding processing stage). Moreover, the novelty of this research relies on the basis of the techno-economic and environmental assessment of biosurfactant productions as a potential key to develop a circular bioeconomy model in developed and developing countries (specifically in the German context). This study assesses the techno-economic and environmental performance of the surfactin production process under different scenarios, considering the European energy crisis of 2021–2022. Moreover, the study compares the techno-economic and environmental behavior of a process with yields obtained from current research data and an optimized yield of *Y*_P/X_ = 1 using a submerged culture of *Bacillus subtilis*. This analysis is addressed to identify bottlenecks and potential solutions to improve process sustainability. Thus, the studied scenarios are (i) A base case with a surfactin-to-glucose yield equal to 0.16 g/g contextualized in 2021 (Sc1), (ii) optimized production with a complete substrate consumption and achieving a product yield of *Y*_P/X_ = 1 (*Y*_P/S_ = 0.30) in 2021 (Sc2), and (iii) a production with a surfactin-to-glucose yield equal to 0.16 g/g contextualized in 2022 (Sc3). Simulation tools such as Aspen v.9.0 and SimaPro v.8.3.3 were used to make all the calculations.

## Methodology

This research focused on the techno-economic and environmental assessment of the surfactin production process using winter wheat as raw material based on the German context. The research study includes the agronomic stage of the winter wheat crop in Germany. The conceptual design of the surfactin production process was done based on published and unpublished data from surfactin production processes (Klausmann et al. 2021). Then, computational tools were used to determine the mass and energy balances of the entire process at a specific scale. The process analysis was done using techno-economic and environmental metrics. The techno-economic assessment was done using financial indicators such as net present value (NPV), payback period (PBP), and surfactin production yield (among others). Supplementary material illustrates some equations used to calculate these financial indicators. Finally, the environmental assessment of the surfactin production process was done by applying the Life Cycle Assessment (LCA) approach using SimaPro v.8.3.3. software.

### Processing scale and process simulation

The surfactin production was designed based on a continuous wheat grain processing of 380 kg/h with the following composition: 12.8% moisture, 12.7% protein, 1.5% ash, 1.5% lipids, and 82.4% starch. This composition was obtained after chemical characterization. Winter wheat grain was used as a raw material since this biomass has been used as a potential source of glucose hydrolysates for different industries and processes in Europe (Salim et al. 2019). Thereby, the Redlich Kwong Equation of State (RK EoS) and the Non-Random Two Liquids (NRTL) activity model were employed to model the liquid and vapor phases in the simulation. Experimental results and theoretical information were used to generate mass and energy balances at the proposed processing scale. The thermodynamic properties of starch and proteins were unavailable in the software database and were therefore obtained from the National Research Energy Laboratory (NREL) (Wooley and Putsche 1996). Indeed, temperature-dependent thermodynamic properties such as vapor pressure, ideal gas heat capacity, heat of vaporization, solid molar volume, solid heat capacity, and liquid heat capacity were added. In addition, non-temperature dependent properties were introduced manually in the Aspen Plus v9.0 software based on the simulation requirements. The added properties were liquid molar volume (RKZTRA), critical temperature (T_c_), critical pressure (P_c_), molecular weight (MW), acentric factor (w), ideal gas heat of formation (DHFORM), ideal gas Gibbs free energy of formation (DGFORM), solid standard enthalpy of formation (DHSFRM), solid standard Gibbs free energy of formation (DGSFRM), and standard enthalpy of combustion (HCOM) (Wooley and Putsche 1996). Specifically, the DHSFRM, DGSFRM, and HCOM properties were estimated as previously reported by Peduzzi et al. (2016). Finally, the surfactin thermodynamic properties were estimated based on a group contribution method (Marrero and Gani 2001).

### Surfactin production process

**Fig. 1:**
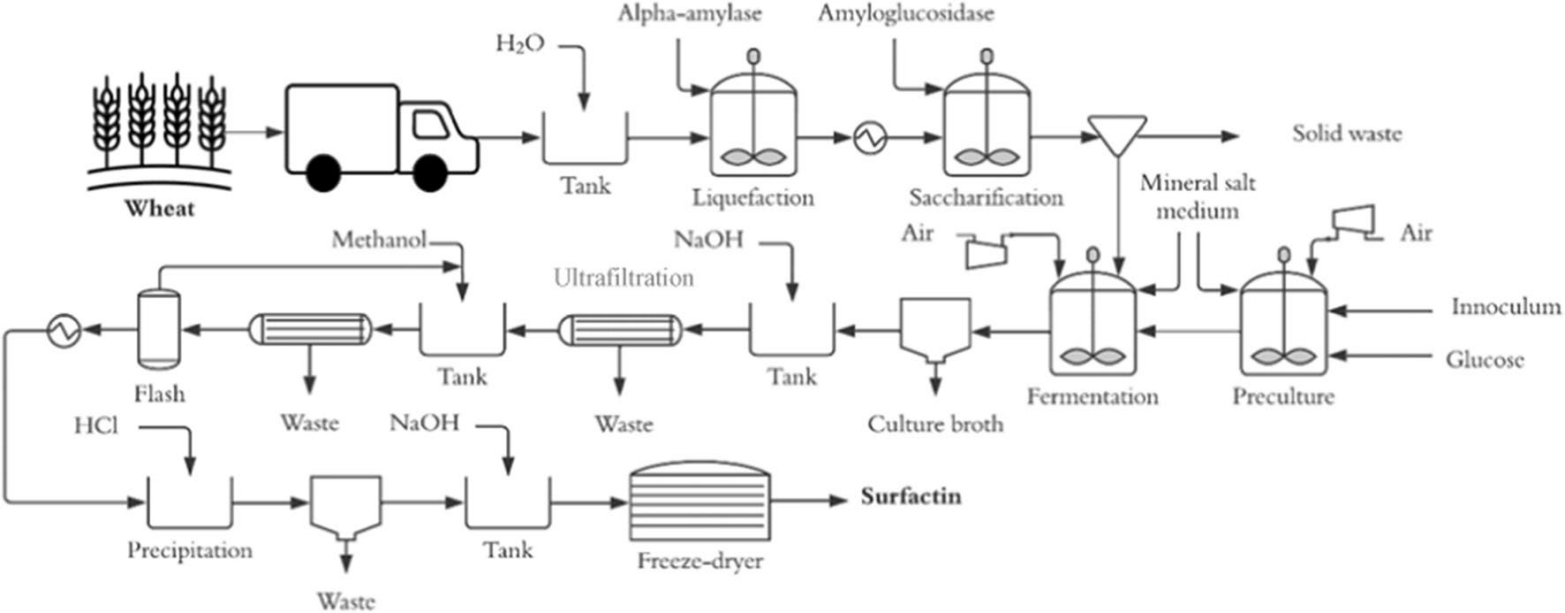
Process flow diagram of the surfactin production process using winter wheat as raw material

Surfactin production was simulated considering a submerged batch cultivation. This process was simulated using software to assess the techno-economic and environmental potential involving different advances in bioprocess engineering and downstream processing. Moreover, surfactin was selected as a key product since there is a wide range of potential applications for this product. The surfactin production process is presented in Fig. 1. The surfactin production process is composed of three stages: (i) raw materials conditioning and glucose production, (ii) surfactin fermentation, and (iii) downstream. Stage I (i.e., raw material conditioning and glucose production) was designed considering typical reports of saccharifying starchy feedstock to glucose (Bello et al. 2021). Finally, Stage II and Stage III were mainly designed based on published and unpublished experimental procedures described by Klausmann et al. (2021). Finally, Stage III was designed based on literature reports (Dolman et al. 2019).

The surfactin production was designed and simulated in the Aspen Plus v9.0 software (Aspen Technology Inc. USA) based on a continuous wheat grain processing of 380 kg/h with the following composition: 12.8% moisture, 12.7% protein, 1.5% ash, 1.5% lipids, and 82.4% starch (composition obtained in the laboratory). Wheat grain was chosen as a crop to obtain glucose through saccharification in Germany (Salim et al. 2019). The simulation considered three scenarios for the surfactin production: (i) A base case with a surfactin-to-glucose yield equal to 0.16 g/g contextualized in 2021 (Sc1), (ii) optimized production with a complete substrate consumption and achieving a product yield of *Y*_*P/X*_ = 1 (*Y*_P/S_ = 0.30) in 2021 (Sc2), and (iii) a production with a surfactin-to-glucose yield equal to 0.16 g/g contextualized in 2022 (Sc3). The yields applied in scenarios 1 and 3 were taken from Klausmann et al. (2021). Scenario 2 used a hypotetically optimized yield based on a *Y*_*P/X*_ = 1 and a surfactin titer of 50 g/L (after 80 h) as reported by Yoneda et al. (2006). For all scenarios, the industrial production scheme was the same as shown in Fig. 1. The surfactin production process was based on Klausmann et al. (2021). The harvested and unground wheat grain was carried to a two-step enzymatic hydrolysis stage to produce glucose hydrolysate following the operating conditions reported by Joelsson et al. (2016). The wheat grain was mixed with sufficient water to 35% dry matter and then liquefied with alpha-amylases at 85 °C (0.5 kg enzyme per ton of wheat grain on a dry basis). Subsequently, the liquefied stream was cooled to 60 °C, and pH was set to 4.2 for saccharification in a second reactor by adding amyloglucosidases at a rate of 1 L per ton of feed on a dry basis (Joelsson et al. 2016). The glucose-rich stream was then fed to a high-cell density fed-batch bioreactor with *Bacillus subtilis* JABs32 (3NA). The preculture and fermentation units were modeled considering the parameter reported by Klausmann et al. (2021) and a high-cell density medium as described by Wenzel et al. (2011). Thereby, an initial batch volume of 12 L was considered, followed by a feeding stage with an additional volume of 8 L (Klausmann et al. 2021). After a cultivation time of 36 h, a biomass of 104.4 g/L and a surfactin concentration of 26.4 g/L were observed based on raw data from Klausmann et al. (2021). The process was scaled up to an industrial scale with a working volume of 65 m^3^ and a total reactor volume of 100 m^3^. After fermentation, the culture broth was removed by centrifugation. The pH of the cell-free product stream was adjusted to 8–9 with the addition of NaOH prior to a sequential ultrafiltration scheme to maximize permeate flux and retentate concentration, achieving 98% lipopeptide recovery (Sen and Swaminathan 2005). Subsequently, methanol is added before the second ultrafiltration stage to improve the diafiltration process by 15% (Coutte et al. 2017). The same recovery yield was assumed for this second ultrafiltration as in the first stage (Isa et al. 2007). This methanol is recovered through evaporation and further recirculated to the ultrafiltration system. The resulting stream is acidified with HCl for precipitation of 97% of the lipopeptide (Chen et al. 2007). The precipitated surfactin is neutralized with NaOH and freeze-dried with 96% dehydration efficiency (Schilling et al. 2014).

### Techno-economic assessment

The mass and energy balances were used to determine indicators such as product yield (PY), mass loss index (MLI), process mass intensity (PMI), and renewable material index (RWI). MLI correlates the waste and product streams while the PMI all the input streams (feedstock and chemical agents) with the product stream. RWI was calculated as the ratio between the feedstock and all the input streams. Moreover, the simulations were analyzed considering the specific energy consumption (SEC) as the energy indicator and correlate the thermal and electric demand of the process. Therefore, the simulations also involved the utility requirement analysis after an energy integration. For the economic assessment, on the other hand, mass and energy balances were also used for the design and costing of the processing units. The capital expense (CapEx) was estimated as direct equipment costs and assembly, instrumentation, civil, piping, and electrical costs. The direct equipment cost was determined using the software of Aspen Process Economic Analyzer v9.0 software (Aspen Technology Inc., USA). The operating expenses (OpEx) were determined as the cost of raw materials, utilities, maintenance, labor, plant overhead, fixed and general, laboratory charges, insurance and taxes, and administrative. Table 1 illustrates the costs of the raw materials and utilities as well as the surfactin market price. Moreover, this information was updated to 2022. The Chemical Engineering Plant Cost Index (CEPCI) of 701.4 for june 2021 and 821.3 for september 2022 was used to calculate the processing and cooling water cost. The US dollar was used as the economic unit, and a straight-line depreciation method was used for the analysis. The interest rates were 0% for 2021 and 2% for 2022 (Economics 2023). Six operators were also assumed with a daily working time of 8 h per shift (three shifts per day) and a pay rate of 11.97 USD/h per employee (Towler and Sinnott 2013; WageIndicator-Foundation 2023).

**Table 1:** Economic parameters used to assess the surfactin process feasibility

Item	Price/cost	Unit	Reference
*Product*			
Surfactin	615	USD/kg	(Kaneka 2023)
*Raw materials*			
Wheat grain	250	USD/ton	(Joelsson et al. 2016)
Alpha-amylase	4.6	USD/kg	
Amyloglucosidases	5.3	USD/kg	
Dipotassium phosphate	0.8	USD/kg	(Czinkóczky and Németh 2020)
Monosodium phosphate	0.5	USD/kg	
Ammonium sulfate	0.19	USD/kg	
Ammonium chloride	0.12	USD/kg	
Ammonia	0.45	USD/kg	
Citric acid	0.6	USD/kg	
Sodium sulfate	0.16	USD/kg	
Magnesium sulfate	55	USD/ton	
Orthophosphoric acid	612	USD/ton	
Sodium hydroxide	0.55	USD/kg	
Hydrochloric acid	0.3	USD/kg	
Methanol	354	USD/ton	
Sodium chloride	50	USD/ton	
*Utilities*			
LP steam	20.8 (2021); 18.5 (2022)	USD/ton	(Kapanji et al. 2021)
MP steam	23.1 (2021); 20.5 (2022)	USD/ton	
Electricity	247.2 (2021); 563.9 (2022)	USD/MWh	

### Environmental life cycle assessment (LCA)

The environmental impact was evaluated using the ISO 14040 methodology (ICONTEC, 2007). The LCA methodology follows four steps: (i) goal and scope definition, (ii) environmental life cycle inventory, (iii) environmental life cycle evaluation, and (iv) results interpretation. These steps are described by the LCA methodology (ICONTEC, 2007). Thereby, the objective is selected initially to define the system boundaries. Afterwards, the scope and functional unit are set. Finally, the most important assumptions of the LCA are described.

#### Goal and scope definition

##### Goal

The goal of the LCA was to compare the environmental impact of surfactin production using winter wheat as raw material in scenarios 1-2. Scenario 3 was not considered since the mass and energy balances are the same if the process is analyzed in 2021 or 2022. No allocations were applied in the LCA since only surfactin is produced. In other words, the aim of the environmental analysis was to analyze the influence of increasing the surfactin production yield on the process environmental performance of the process. Thus, the environmental assessment seeks to answer the following question: How would the surfactin production process perform if (close to)-ideal yields were assumed e.g. optimized *Y*_P/X_ of 1?

##### Scope

A cradle-to-gate approach was chosen as scope. Thus, the environmental analysis involves the winter wheat grain production in Germany and the surfactin production process using *Bacillus subtilis* as a microorganism. The LCA involves the agronomic data reported by The Board of Trustees for Technology and Construction in Agriculture (KTBL) database (KBTL 2023). Fertilizers, herbicides, pesticides, and machinery used in wheat harvesting were considered. The agronomic stage (i.e., wheat crop and harvesting) was subdivided into the following stages: (i) tillage, (ii) seeding, (iii) plant protection and fertilizing, and (iv) harvesting. The crop yield was set at eight (8) tonnes per ha (Anthoni et al. 2004).

##### Functional unit

Two functional units were considered in the LCA. The first functional unit was 1 tonne of winter wheat produced in the agronomic stage. The second functional unit was 1 tonne of surfactin produced in the fermentation process. These functional units were selected in line with the system boundaries to assess the environmental impact of the agronomic stage and the surfactin production process as part of the LCA goal. The functional units were selected to analyze in a better way the impact of each stage on the global environmental performance of the process and identify potential hotspots. 

##### System boundaries

**Fig. 2:**
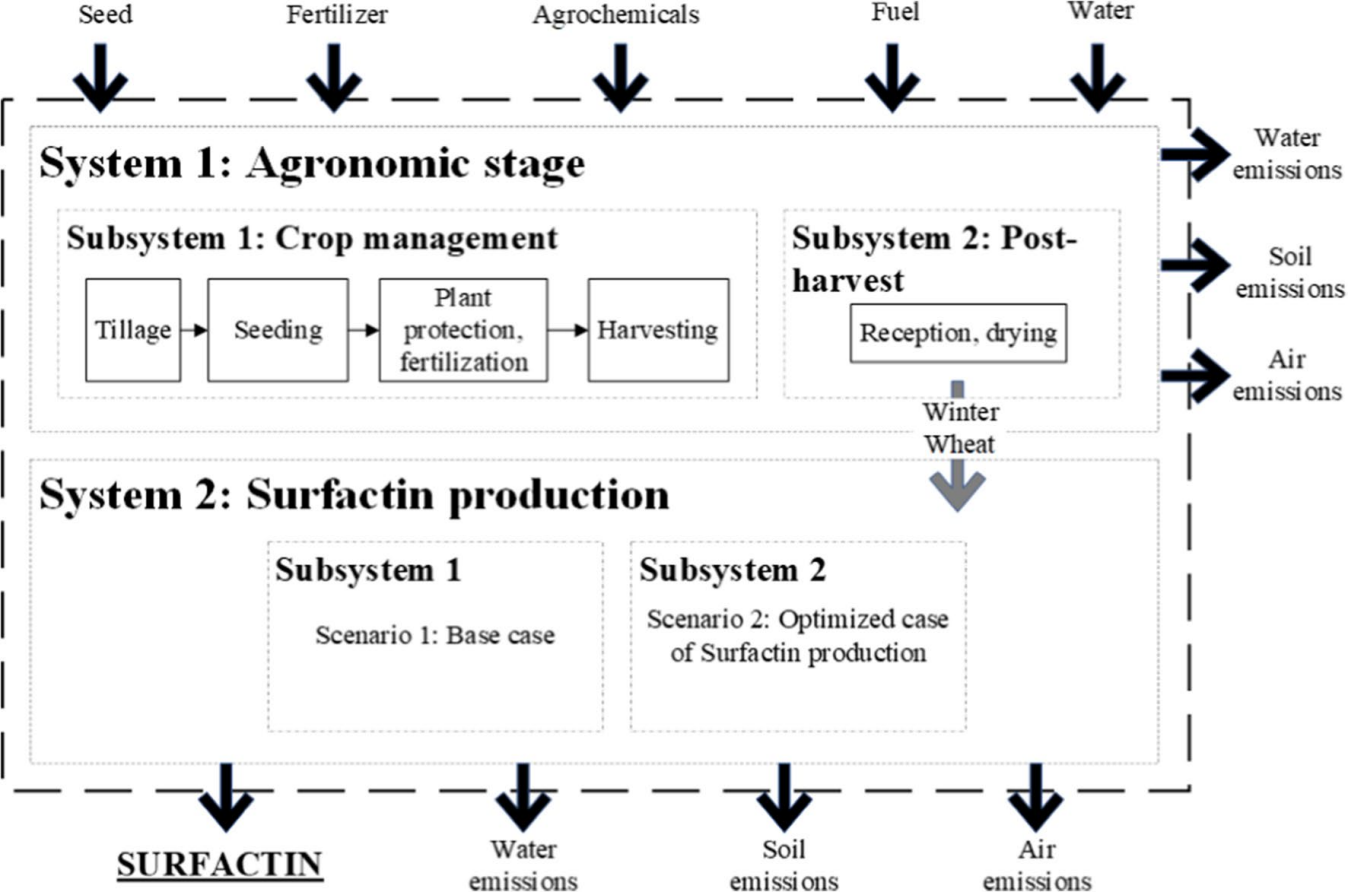
System boundaries of the surfactin production process considering the winter wheat production in Germany

The LCA systems, subsystems, and activities are presented in Fig. 2. The surfactin production process using winter wheat as raw material is presented based on the three scenarios proposed in previous sections. Global inputs and outputs are presented. Surfactin is a unique product from the proposed process.

The first system is related to the agronomic stage. This system was subdivided into two subsystems: (i) crop management and (ii) post-harvest. The activities involved in each subsystem are also presented. Quantitative information on these activities is presented in the life cycle inventory. The winter wheat produced in the agronomic stage is sent to the biosurfactant production process. The most important difference between subsystems 1 and 2 is related to the surfactin production yield since the mass and energy balances of the process are not affected by the European energy crisis of 2021–2022. Thus, two environmental impact results are reported. Finally, emissions to air, water, and soil in both systems were considered.

#### Environmental life cycle inventory (LCI)

The LCI was done based on the information reported by The Board of Trustees for Technology and Construction in Agriculture (KTBL) database (KBTL 2023). The first system (Agronomic Stage) involves crop management and post-harvest subsystems. In crop management, tillage, seeding, plant protection, fertilization, and harvesting are carried out. In tillage, 400 kg of phosphorus-potassium fertilizer is added per hectare with a centrifugal spreader. Then, plowing is carried out with a reversible plow. Planting is carried out with a mechanically mounted seeder where 180 kg of seeds are added per hectare. For winter wheat protection and fertilization activity, 0.9 kg of isoproturon (herbicide) is added with 300 L of water for weed control. Four months after sowing, the first crop fertilization with calcium ammonium nitrate is carried out with a mechanical centrifugal spreader at 200 kg/ha. In the fifth month, a phytosanitary sprayer consists of 260 kg of calcium ammonium nitrate, 2 L of ethephon, and 300 L of water per hectare of winter wheat. Starting the sixth month of cultivation, fungicidal (0.75 L of bitertanol) is added. In the eighth month of the crop, the third dose of fertilizer (160 kg of calcium ammonium nitrate per hectare) and fungicide (0.75 L of bitertanol) is added. The fourth activity of the crop management subsystem is the harvest, where at the beginning of the ninth month, a 200-kW combine is used. The crop yield is 8 tons of winter wheat per hectare (Anthoni et al. 2004). The second-grain reception subsystem includes the drying of winter wheat with machinery continuously. Finally, 3 tons of calcium carbonate are added for every 6 tons of winter wheat (Anthoni et al. 2004). The inventory of system one is presented in Table 2

**Table 2:** Life cycle inventory of the agronomic stage (i.e., winter wheat production) based on (KBTL 2023)

Subsystem	Activity	Time (month)	Inputs		
			Item	Value	Unit/ha
Crop management	Tillage	1	Fuel-small truck	0.8	L
			Fertilizer 1^a^	50	kg
			Fuel-tractor 45 kW	0.69	L
	Seeding	1	Seed	22.5	kg
			Fuel-tractor 45 kW	30.08	L
	Plant protection and fertilization	9	Herbicide 1^b^	11.25	g
			Fertilizer 2^c^	25	kg
			Fertilizer 3^c^	32.5	kg
			Growth regulator 1^d^	250	mL
			Water	37.5	L
			Fuel-sprayer	112.5	L
			Fungicide 1^e^	9.38	mL
			Fuel-shaker	4.77	L
Post-harvest	Grain processing	1	Lime	500	g
			Fuel-transport^f^	138.75	mL
			Fuel-tractor front loader	1.25	mL
			Fuel-centrifugal spreader	20	mL
			Fuel-swivel plow	6.72	L
			Fuel-seedbed combination	651.25	mL

^a^Phosphorus-potassium (PK-18% P_2_O_5_, 10% K_2_O)

^b^Isoproturon

^c^Calcium ammonium nitrate (27% N)

^d^Ethephon

^e^Bitertanol

^f^Three-way tipping trailer

The LCI related to air, water, and soil emissions were estimated based on the diesel fuel combustion in internal combustion engines (ICE) and the use of agrochemicals (i.e., fertilizers, pesticides, and herbicides). The emissions were estimated using IPCC methodologies (De Klein et al. 2006; IPCC. 2006). The emissions to air caused by diesel combustion are presented in Table 3, while the emissions caused by the agrochemicals use are presented in Table 4. All the values reported in Table 3 can be recalculated per tonne of surfactin, knowing the surfactin yield per kg of winter wheat. Moreover, the values in Table 4 can be recalculated per tonne of surfactin, considering the crop yield and production yield. Carbon sequestration was not considered.

**Table 3:** Emissions from diesel combustion related to the total fuel demand

Released compound	Emission (kg kg^−1^ fuel)	Total emission (kg/ha)
SO_2_	0.01071	5.63
NO_x_	0.04286	22.53
CO_2_	3.47857	1828.65
CO	0.00679	3.57
VOC	0.00393	2.07
N_2_O	0.00071	0.38
Pentane	0.00036	0.19
NMVOC	0.00004	0.02
CH_4_	0.00014	0.08
Soot	0.00129	0.68

**Table 4:** Summary of total emissions of agrochemical and fertilizer use

Emission	Compound	Value	Unit
To the air	N_2_O total	6009.2	g/ha
	NH_3_	4204.0	g/ha
	CO_2_	132.0	g/ha
To the water	NO_3_^−^	51,931.2	g/ha
	PO_4_^−3^	245.3	g/ha
	P	1.4	g/ha
To the soil	P	1.7	g/ha

The mass and energy balances after the simulation procedure were considered input information in the life cycle inventory of the surfactin production process. The energy (electricity) required in the surfactin-produced process was assumed to be produced by the German energy grid. Then, the energy mix considered for the electricity production in Germany in 2021 was lignite (18.78%), coal (9.29%), nuclear energy (11.79%), natural gas (15.38%), oil (0.80%), wind (19.48%), solar PV (8.39%), biomass (7.49%), hydropower (4%) (Appunn et al. 2023). On the other hand, the thermal energy required by the process was assumed to be provided by the boiler using natural gas as fuel. The final assumption taken into considerartion to perform the LCA was related to the releases done during the surfactin production process. Indeed, this study did not consider the CO_2_ emissions during the fermentation.

#### Life cycle impact assessment

The software SimaPro v 8.3 was applied for the environmental life cycle evaluation utilizing the database EcoInvent 3. Calculations in the software were set to follow the ReCiPe midpoint (Hierarchist version) approach.  The characterization factor of climate change is the global warming potential, based on the IPCC 2013 report. 100 years were set as time horizon and used for the Hierarchist perspective. Climate-carbon feedbacks are included for non-CO_2_ greenhouse gases in this perspective. Climate change (kg CO_2_-eq), water depletion (m^3^), and fossil depletion (kg oil-eq) were some of the midpoint impact categories assesed by the described method.

#### Results interpretation

The interpretation of the LCA results is done to identify the most important factors affecting the environmental performance of the process. Moreover, the LCA results are used to change current practices in the agronomic stage as well as identify the most critical factors in the surfactin production process. Finally, conclusions, limitations, and recommendations can be made for future work and comparisons. 

## Results and discussion

### Techno-economic assessment of the surfactin production process

Table 5 summarizes the results of mass indicators of surfactin production of the base case and the ideal process. For the base case, 35.1 kg/h of lyophilized surfactin was produced, and the PY corresponds to 36.3% of the optimized process. Surfactin yields prior to separation for scenario 1 agree with some literature reports using *Bacillus subtilis* and glycerol as carbon source or even higher than surfactin based on crude glycerol from different sources such as biodiesel, soap, and stearin (De Faria et al. 2011; Janek et al. 2021). Czinkóczky and Németh (2020) have reported overall yields of 0.143 kg surfactin per kg glucose for a full-scale facility. The separation methodology explains the differences between the results obtained since the authors only report precipitation, centrifugation, and spray drying, obtaining surfactin at a purity of 89.4% (Czinkóczky and Németh, 2020). The surfactin yield decreased through each operating unit due to physicochemical limitations of the process stream or inefficiencies of the processing units. Surfactin losses exceeded 30% in the base case, especially in the ultrafiltration stages, accounting for more than half of the total. Regarding the PMI, the surfactin production of the base case refers to the production of fine chemical compounds since it exceeds the threshold of bulk processes (50 kg feedstock per kg product) (Tobiszewski et al. 2015). This result has been also reported for other biosurfactans such as the sophorolipid from sugarcane bagasse (Elias et al. 2021), rhamnolipid from oil palm empty fruit bunches (Efendi et al. 2023), and lichenysin and surfactin from glucose (Czinkóczky and Németh 2020). Note that the ideal process has a different trend, achieving a bulk process classification (see Table 5). It was also observed that the ML_I_ has similar values to the PMI for both scenarios, inducing that surfactin production demands high use of inputs such as chemical compounds and process water. For example, the base case requires 8.97 L of processing water per kg of feedstock, while the ideal process requires only 5.47 L/kg. However, there is a substantial change in these indicators between scenarios, and the base case corresponds to 23% of the ideal process. Regarding the RM_I_, it was determined that the base case requires a higher usage of inputs to separate a smaller amount of product, which was expected for the neutralization and methanol separation stages. For the energy indicator analyzed, there is a slight increase in energy requirement between the different scenarios, mainly LP steam and electricity, as shown in Table 6. The MP steam and refrigerant were the same for both scenarios. The refrigerant was determined for a 165-kWh freeze dryer with sufficient working capacity for the base case and ideal process.

**Table 5:** Mass and energy indicators

Indicator	Units	Scenario 1	Scenario 2
P_Y_	kg/100 kg feedstock	9.24	25.43
ML_I_	kg/kg surfactin	114.95	27.36
PMI	kg/kg surfactin	115.95	28.36
RM_I_	kg feedstock/ton	93.36	138.68
SEC	MJ/kg	7.70	7.97

**Table 6:** Energy utility yields for the proposed scenarios

Utility	Units	Scenario 1	Scenario 2
LP steam	kg/kg feedstock	1.10	1.12
MP steam	kg/kg feedstock	0.06	0.06
Cooling water	L/kg feedstock	554.84	670.44
Refrigerant	kg/kg feedstock	0.84	0.84
Electricity	kWh/kg feedstock	1.31	1.41

Table 7 summarizes the analysis of economic parameters of surfactin production for 2021 and 2022. For both scenarios, there was no CapEx variation, and it was determined that the direct equipment cost represented 57% of the capital cost, where the freeze dryer was the most expensive processing unit (1.01 M-USD) with a processing capacity of 500 kg per 16 h loads. Instrumentation costs (1.34 M-USD) were mainly attributed to the total number of transmitters for the entire processing system, distributed as 36 pressure, 18 level, 30 temperature, and 46 flow transmitters. The remainder of CapEx was for civil work (i.e., foundation and pilling, dikes, and pipe rack) and accounted for 9% of the total, followed by piping (5%), electrical, and firefighting (1%) costs. Regarding the OpEx, raw materials, utilities, and labor were the most representative of both scenarios. The cost of winter wheat represented nearly 50% of raw materials, followed by methanol. In contrast to the CapEx, the European energy crisis contextualized to Germany drastically affected OpEx, increasing by 21.1% and mainly attributed to utilities. The utility cost distribution was mainly for refrigerant (1.26 M-USD/year) and electricity (1.08 M-USD/year) by 2021, while the costs changed to 2.45 M-USD/year for electricity (an increase of 1.38 M-USD/year) and 1.28 M-USD/year for the refrigerant by 2022. Consequently, the production cost and gross income were negatively affected (see Table 4). However, the high revenue from surfactant sales buffers the 2022 energy consequences and does not alter economic viability since the NPV of the 2021 and 2022 scenarios corresponds to 70% and 38% of the ideal surfactin process. Both scenarios demonstrated a high economic viability, with payback possible in less than 1 year of start-up. The NPV after 20 years of useful life decreased by 43% in 2022 as the cost of electricity drastically affects demanding processing units such as freeze dryers or compressors. Despite this NPV decrease, the 2022 scenario offers a profit margin above 100%. Due to this economic feasibility, a sensitivity analysis of the process cash flows was performed to determine the minimum processing scale for economic feasibility (MPSEF). This analysis aimed at minimizing the processing scale, affecting the input and output flows as well as CapEx due to the equipment size reduction, until an NPV of zero (NPV = 0) at 20 years of lifetime. The MPSEF for the 2021 base case was 4.13 kg/h, corresponding to a cultivation area of about 4.7 ha for a yield of 7.7 tons/ha of winter wheat (Macholdt and Honermeier 2017), while 5.2 ha represents the 2022 process. A sensitivity analysis concerning the selling price of surfactin can also be proposed, whose value may decrease to a minimum price (for NPV = 0) of 29 USD/kg and 31 USD/kg for the 2021 and 2022 scenarios, respectively.

**Table 7:** Summary of economic comparison of surfactin production in 2021 and 2022

Economic parameter	Scenarios comparison	
	Scenario 1	Scenario 3
CapEx (M-USD)	8.42	
OpEx (M-USD/year)	5.85	7.09
Raw materials	1.75	1.77
Utilities	2.60	3.94
Labor	0.82	0.92
Maintenance	0.58	
Others^*^	1.82	1.72
Production cost (USD/kg)	26.41	30.52
Revenues (M-USD/year)	189.25	
Gross income (M-USD/year)	181.12	179.86
Payback (years)	0.76	0.98
NPV (M-USD/year)	2468.98	1406.66
MPSEF (kg/h)	4.13	4.57

^*^Fixed, general, plant overhead, laboratory charges, insurance and taxes, and administrative costs

The study of production costs is fundamental for the development of a biotechnological process that allows optimizing production operations, estimating overall profit margins, minimizing costs, and ensuring product continuity in the market. The production of surfactin from wheat straw seems to be attractive to investors or sponsors because of its opportunity for industrial-scale application based on the conceptual design approach and the techno-economic assessment. Although the cost of production may be high in contrast to synthetic surfactants (Soares da Silva et al. 2017), its interest lies in different cost–benefit implications which have also been reviewed by other authors (Wang et al. 2020). Moreover, the development of biosurfactants has not only been driven by recent environmental legislation but also by the increasing expansion of the market and producers. Previous reports have mentioned a compound annual growth rate of over 5.5% with projections of up to 2.5 M-USD by 2026 (Longati et al. 2023). Thus, the efforts to produce biosurfactants from lignocellulosic residues should be increased on an annual basis to increase commercial availability and make the production cost competitive with synthetic surfactants. In fact, biosurfactant production companies have been identified depending on the industrial application (Farias et al. 2021). A target market analysis is essential for the installation and start-up of industrial-scale production. Biosurfactants for cosmetic, personal care, or pharmaceutical use, such as surfactin, should be considered small-scale processes due to the high costs and technological complexity of the downstream stages. For industrial companies where the purity of the biosurfactant should not be so high (i.e., for cleaning and hygiene products), fermentations through crude broths using mixtures of carbon sources have been proposed (Farias et al. 2021).

### Environmental life cycle assessment results

The environmental LCA results are divided into two subsections related to the environmental impact of winter wheat production and the whole surfactin production process. The overall environmental impact per functional unit is presented in the following tables and figures.

#### Winter wheat production

Winter wheat production was analyzed as a potential source of glucose to produce surfactin. An analysis of the environmental impact associated with the crop and harvesting is necessary to know the influence of this system on the whole environmental performance of the surfactin production process (see Fig. 1). The results of the environmental evaluation are presented in Table 8**.**

**Table 8:** Environmental life cycle assessment results of the winter wheat production in Germany

Impact category	Value	Units	Impact category	Value	Units
Climate change	4.81E-01	kg CO_2_ eq	Terrestrial ecotoxicity	7.9E-07	kg 1,4-DB eq
Ozone depletion	5.58E-11	kg CFC-11 eq	Freshwater ecotoxicity	2.9E-06	kg 1,4-DB eq
Terrestrial acidification	1.00E-04	kg SO_2_ eq	Marine ecotoxicity	2.3E-05	kg 1,4-DB eq
Freshwater eutrophication	1.10E-05	kg P eq	Ionizing radiation	1.2E-04	kBq U235 eq
Marine eutrophication	1.50E-03	kg N eq	Agricultural land occupation	3.7E-03	m2a
Human toxicity	1.01E-03	kg 1,4-DB eq	Water depletion	2.1E-03	m^3^
Photochemical oxidant formation	7.92E-05	kg NMVOC	Metal depletion	3.7E-05	kg Fe eq
Particulate matter formation	2.23E-05	kg PM10 eq	Fossil depletion	1.9E-02	kg oil eq

Two of the most significant categories are climate change and water depletion. Climate change can be related to the carbon footprint of winter wheat production, while water depletion can be related to the blue water footprint required in the harvest activities. The carbon footprint calculated for winter wheat production is 0.48 kg CO_2_-eq/kg. This result is lower than the values reported by He et al. (2023). These authors reported a carbon footprint of 0.60 kg CO_2_-eq/kg of winter wheat harvested in China. Similar results can be found in the literature. For instance, Luo et al. (2021) reported a range between 0.40 and 0.60 kg CO_2_-eq/kg winter wheat for the carbon footprint at different crop conditions. Hirschfeld et al. (2008) reported the carbon footprint of winter wheat in 0.40 kg CO_2_-eq/kg applying conventional agricultural practices in Germany. Thus, the results obtained based on the LCI presented in Table 2, Table 3, and Table 4 can be used to analyze different productive schemes based on winter wheat. The activity with the highest impact on the carbon footprint was plant protection and fertilization. This activity consumes the highest amount of diesel fuel. Moreover, this activity introduces the use of fertilizers, pesticides, and herbicides, which harm the environment due to air, water, and soil emissions since nitrogen-based fertilizers are commonly associated with high GHG emissions (Bonales-Revuelta et al. 2022). The plant protection and fertilization activity accounts for more than 87% of the total carbon footprint based on the present research results. This share is similar to the value reported by Luo et al. (2021). Indeed, these authors attributed 79% of the total carbon footprint. Machinery use and agrochemical transportation are the most critical bottlenecks in this crop. Strategies to reduce the environmental impact of winter wheat production are related to decreasing the use of chemical fertilizers and reducing the use of oil-based fuels. These strategies can be performed using bio-based fertilizers derived from compost and biofuels such as biodiesel. A carbon footprint reduction higher than 50% can be reached by applying the abovementioned strategies (Hirschfeld et al. 2008). The other impact categories reflect a low environmental impact.

#### Surfactin production process

**Fig. 3:**
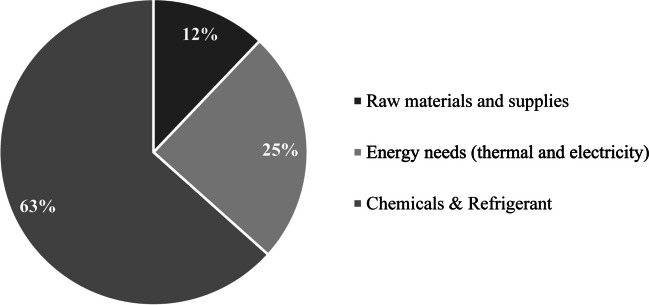
Contribution share to the total carbon footprint of the surfactin production process of the most important process inputs

The surfactin production process results are presented in Table 9**.** Scenario 1 and scenario 2 have differences in all impact categories. The carbon footprint and ozone depletion decrease 2.7-fold times when the fermentation surfactin-to-glucose yield is decreased by 50%. These results evidence the high impact of this variable on the environmental performance of the process. In this way, the raised question in the goal of the environmental analysis can be solved. The environmental performance of the process can be better if the yields of the surfactin production process are optimized (i.e., *Y*_*P/X*_ = 1 and *Y*_P/S_ = 0.30). Thus, more efforts should be done to improve glucose conversion to surfactin analyzing different perspectives such as bioprocess engineering, process configuration, and media composition. Nevertheless, the carbon footprint results should be analyzed considering the most influential factors. Figure 3 shows the contribution share to the carbon footprint based on the surfactin process inputs.

**Table 9:** Environmental impact categories related to the production of 1 kg of surfactin

Impact category	Scenario 1	Scenario 2	Units
Climate change	4.25E + 01	1.57E + 01	kg CO_2_ eq
Ozone depletion	5.93E-06	2.17E-06	kg CFC-11 eq
Terrestrial acidification	1.84E-01	6.70E-02	kg SO_2_ eq
Freshwater eutrophication	1.43E-02	5.53E-03	kg P eq
Marine eutrophication	2.18E-02	7.99E-03	kg N eq
Human toxicity	1.39E + 01	5.25E + 00	kg 1,4-DB eq
Photochemical oxidant formation	6.43E-02	2.36E-02	kg NMVOC
Particulate matter formation	6.23E-02	2.27E-02	kg PM10 eq
Terrestrial ecotoxicity	8.02E-03	2.91E-03	kg 1,4-DB eq
Freshwater ecotoxicity	2.56E-01	9.78E-02	kg 1,4-DB eq
Marine ecotoxicity	3.23E-01	1.22E-01	kg 1,4-DB eq
Ionizing radiation	2.98E + 00	1.10E + 00	kBq U235 eq
Agricultural land occupation	4.15E-01	1.54E-01	m2a
Water depletion	1.23E-01	4.55E-02	m^3^
Metal depletion	1.20E-02	4.37E-03	kg Fe eq
Fossil depletion	6.21E-01	2.26E-01	kg oil eq

Refrigerant, energy needs, and raw materials contribute 63%, 25%, and 12% to the total carbon footprint. Only one distribution graph is presented since scenario 1 and scenario 2 have similar trends (i.e., the difference between the contribution shares is not higher than 2%). These results allow elucidation of the high impact related to the use of chemicals and refrigerants in the surfactin downstream. The acid precipitation methods have been used as a preliminary method to separate lipopeptides (Dolman et al. 2019). Nevertheless, an organic solvent is needed to recover surfactin from the precipitated solids (i.e., not only surfactin precipitates by changing pH). These two steps must be studied to reduce the amount of inorganic acid and solvent used since a constant flow of these materials is needed in production. On the other hand, the temperature decreases by using ammonia as a refrigerant to increase product purity has the highest impact on the process since it contributes more than 55% to the total carbon footprint of the process. In this way, if ammonia is avoided, a significant reduction in the environmental impact can be reached. Indeed, the surfactin production process carbon footprint can be reduced to 15.2 and 5.77 kg CO_2_ eq/kg surfactin, considering scenario 1 and scenario 2, respectively.

The second most important factor contributing to the high carbon footprint is the process energy demand. This item contributes 25% to the total environmental impact. Thus, strategies to reduce steam and electricity consumption must be addressed. A more in-depth look at the energy supply process allows for elucidating the electricity demand as the most critical factor since the electricty provided by the German energy grid contributes 82% to the total carbon footprint . In other words, the electricty production is one of the most important hotspots to be analyzed based on the electricty demand. Similar trends were reported by Kopsahelis et al. (2018). These authors reported a high carbon footprint of the sophorolipids and rhamnolipids due to the high energy demand of the process in Greece. Consequently, an alternative to reduce the environmental impact related to the energy demand is integrating a cogeneration unit able to supply 100% of the energy needs of the surfactin production process. This statement can be true because the surfactin production process can be designed at a small scale based on the economic results. Therefore, the concept of biorefinery gains importance due to the generation of a series of high value-added products (e.g., lignin derivatives, organic acids, alcohols, esters, among others) and energy vectors (e.g., biogas, syngas, and hydrogen) addressed to the minimization of environmental impact by releasing lower amounts of CO_2_ than conventional energy supply alternatives (Lopes et al. 2019; Sharma et al. 2023).

The calculated carbon footprint of the surfactin production process can be affected by the carbon dioxide emissions produced during the fermentation. Indeed, different strains of *Bacillus subtillis* can produce carbon dioxide due to the metabolic pathway. Moreover, CO_2_ production during surfactin fermentation can be controlled by media composition. Valdés-Velasco et al. (2022) reported the C-mol yields of surfactin and carbon dioxide using different strains in submerged fermentations (i.e., the authors reported a C-mol yield of 0.49 mol-C CO_2_/mol-C of glucose and 0.01 mol C of surfactin/mol-C of glucose). The highest carbon dioxide yield was 25 g/g of surfactin. Then, this high carbon dioxide yield can become an issue in the surfactin production process if it is not controlled. Carbon footprint can increase more than 60% by considering carbon dioxide emissions. Thus, a comprehensive characterization of the mass balances of the surfactin production process is needed to improve the quality of the process LCI.

The surfactin environmental results agree with other literature reports related to biosurfactant production. Longati et al. (2023) reported a carbon footprint between 0.9 and 6.1 kg CO_2_ eq/kg of biosurfactant produced. Moreover, Elias et al. (2021) reported a carbon footprint of 9.55 and 17.1 kg CO_2_ eq/kg of sophorolipids when using ultrafiltration and solvent extraction, respectively. These results demonstrate the influence of the downstream processing on the environmental impact of the process due to the use of solvents increasing the carbon footprint by almost 100%. The same trend can be identified in the results of this research study, where ammonia is the most critical factor in environmental performance. Therefore, the analysis of other separation technologies is needed. The carbon footprints presented and discussed are higher than the current environmental impact caused by chemical surfactant production. Nevertheless, the technological readiness level of biosurfactant production is lower compared with the current development related to chemical surfactant production. This “low development” is evidenced in the wide variety of downstream configurations proposed in patents and research papers, which must be evaluated from a process engineering perspective and assessed as a reliable and feasible option. Even so, biosurfactants such as surfactin can be profiled as a potential option when more mature technologies are implemented at the industrial level.

The water footprint of the surfactin production process was 123 L/kg of surfactin and 46 L/kg of surfactin in scenario 1 and scenario 2, respectively. The water demand of the surfactin production process is attributed to the inoculum preparation and fermentation process (i.e., submerged fermentation). The difference between scenario 1 and scenario 2 related to the water consumption is explained by the water needs in the downstream processing since more amount of product requires more supplies in this stage. In this sense, the total water footprint of the surfactin production process including the winter wheat stage was 125 L/kg and 48 L/kg of surfactin, respectively. Then, the highest water consumption is present in the surfactin production process. The results are difficult to compare with literature reports of the same product since few papers have addressed the environmental assessment of the surfactin production process. Nevertheless, the water consumption of bioprocesses is similar for different cases (e.g., bioethanol, sorbitol, xylitol) (Zaky et al. 2020). Indeed, the water demand is centralized in the fermentation stage. Low substrate concentrations imply high water consumption, but high substrate concentrations require lower amounts of water (Boodhoo et al. 2022). Finally, scenario 2 presented the best environmental performance since most product was produced under the optimized conditions. Then, an improvement of the process performance (i.e., carbon source conversion and separation) increases the environmental performance of the process.

The sustainability of the surfactin production process can be analyzed based on the technical, economic, and environmental information given in this research study. This process presents a good economic performance considering a minimum selling price between 29 and 31 USD/kg. On the other hand, the carbon and water footprint of the process is high compared to other processes. The high values of these indicators are based on the total amount of produced surfactin. In this way, the surfactin production process (i.e., biosurfactants) can perform well as long as the process incomes are high. Nevertheless, several authors have reported the unfeasibility of biosurfactant production. The most critical aspects related to the unfeasiblity of the surfactin production process are related to the fermentation process (e.g., increase yields based on mass transfer and genetical modifications) and downstream processing. Despite this, shareholders such as BASF SF and Evonik are strengthening agreements to encourage worldwide biosurfactant production. Thus, these kinds of products can offer good opportunities in the future to replace oil-based products in a circular bioeconomy context.

## Conclusions

The technical assessment demonstrated that an increase of fourfold times of the global process mass inefficiency is achieved when reducing 50% the surfactin-to-glucose yield. The economic assessment demonstrated the economic feasibility of surfactin production using glucose as feedstock obtained from winter wheat. A sensitivity analysis of the surfactin-to-glucose yield verified the low effect of this variable on the economic performance since positive net present values were reached at the proposed scale (380 kg/h). The energy crisis in Europe 2021–2022 did not have a high effect on the economic performance since the surfactin production process is feasible at small scales. The minimum surfactin selling prices were between 29 and 31 USD/kg. A 42.46 kg CO_2_eq/kg carbon footprint of surfactin was found when the surfactin-to-glucose yield is 0.30 (i.e., *Y*_P/S_ = 0.16 g/g). Specifically, the net carbon footprint was 37.25 kg CO_2_eq/kg of surfactin, leaving aside the emissions caused by the winter wheat production. An increase in the fermentation yields (i.e., *Y*_P/X_ = 1 and *Y*_P/S_ = 0.30) reduces the environmental impact until 15.2 kgCO_2_eq/kg. Finally, the downstream processing was the most critical stage in the production process since the use of chemical reagents (e.g., hydrochloric acid and methanol) and refrigerant (i.e., ammonia) contribute 63% to the total carbon footprint. The energy demand of the process was also identified as a critical factor because it contributed 25% to the total carbon footprint.

## Supplementary Information

Below is the link to the electronic supplementary material.

Supplementary file1 (DOCX 22 KB)

## Data Availability

The datasets used and/or analyzed during the current study are available from the corresponding author on reasonable request.
