# Supplementary material for: Assessing the feasibility and sustainability of a surfactin production process: a techno-economic and environmental analysis
Source: Environ Sci Pollut Res Int. 2024 Apr 9;32(48):27699–714. doi: 10.1007/s11356-024-32217-0 (PMC12696097; doi:10.1007/s11356-024-32217-0)
Supplement: Supplementary file 1 — Supplementary file1 (DOCX 22 KB) [file 11356_2024_32217_MOESM1_ESM.docx]

**Supplementary Information**

**Detailed economic parameters for simulation and analysis**

Some parameters, equations and calculations for the economic analysis are described above:

- Working capital: 15% of total capital investment
- Operator hours per day:

$$\frac{Operator hours}{day}=A\times{Plant Capacity \left[ \frac{tons of product}{day} \right]}^{B}$$

Where A and B are constants calculated with potential regression, based on (Peters & Timmerhaus, 1991)

- Operating supervision: 15% of operating labor
- Laboratory charges: 20% of operating labor
- Taxes (property): 2% of fixed capital investment
- Insurance: 1% of fixed capital investment
- General Costs: 20% of labor, supervision and maintenance
- Fixed charges: Sum of taxes and insurance
- Plant overhead: 60% of labor, supervision and maintenance
- Administrative cost: 25% of plant overhead
- Start-up cost: 10% of fixed capital cost
- Annual depreciation:

$$AD=\frac{IR\times\left( 1+IR \right)^{Lifetime}\times(SUC+FCC)}{{((1+IR)}^{Lifetime})-1}$$

Where, AD: Annual depreciation (mUSD/year); IR: Interest rate (%); Lifetime: 20 years; SUC: Start-up costs; and FCC: Fixed capital costs (mUSD/year)

- Profit margin:

$$Profit margin \left( \% \right)=\frac{Sale price-production cost}{Sale price}*100$$

- Gross income:

$$Gross income=Revenues-OpEx$$

- Net Present Value (NPV):

$$NPV=\sum_{t=1}^{n} \frac{{NCF}_{t}}{\left( 1+k \right)^{t}}-I_{0}$$

Where, I_0_: Initial capital investment; NCF_t_: Net cash flow; k: Discount rate or opportunity rate; and n: number of periods.

**References**

Peters, M. S., & Timmerhaus, K. D. (1991). *Plant design and economics for chemical engineers*. McGraw-Hill.
